# Tai Chi exercise and functional electrical stimulation of lower limb muscles for rehabilitation in older adults with chronic systolic heart failure: a non-randomized clinical trial

**DOI:** 10.1590/1414-431X20198786

**Published:** 2019-11-21

**Authors:** Yi Hao, Long Zhang, Zhenhua Zhang, Lin Chen, Ning He, Shuai Zhu

**Affiliations:** 1 Department of Cardiac Surgery, Beijing Luhe Hospital, Capital Medical University, Beijing, China; 2 Department of Cardiology, Beijing Luhe Hospital, Capital Medical University, Beijing, China

**Keywords:** Geriatric, Heart failure, Kansas City Cardiomyopathy Questionnaire, Functional electrical stimulation of lower limb muscles, Tai Chi exercise

## Abstract

Exercise-based training decreases hospitalizations in heart failure patients but such patients have exercise intolerance. The objectives of the study were to evaluate the effect of 12 weeks of Tai Chi exercise and lower limb muscles' functional electrical stimulation in older chronic heart failure adults. A total of 1,084 older adults with chronic systolic heart failure were included in a non-randomized clinical trial (n=271 per group). The control group did not receive any kind of intervention, one group received functional electrical stimulation of lower limb muscles (FES group), another group practiced Tai Chi exercise (TCE group), and another received functional electrical stimulation of lower limb muscles and practiced Tai Chi exercise (FES & TCE group). Quality of life and cardiorespiratory functions of all patients were evaluated. Compared to the control group, only FES group had increased Kansas City Cardiomyopathy Questionnaire (KCCQ) score (P<0.0001, q=9.06), only the TCE group had decreased heart rate (P<0.0001, q=5.72), and decreased peak oxygen consumption was reported in the TCE group (P<0.0001, q=9.15) and FES & TCE group (P<0.0001, q=10.69). FES of lower limb muscles and Tai Chi exercise can recover the quality of life and cardiorespiratory functions of older chronic heart failure adults (trial registration: Research Registry 4474, January 1, 2015).

## Introduction

The prevalence of heart failure in the aging Chinese population is increasing. Patients hospitalized with heart failure have a risk of depression and high-cost care (1). Heart failure patients have exercise intolerance (2). Exercise-based training improves survival and decreases hospitalizations in chronic heart failure (CHF) patients (3). Exercise is a non-pharmacological but comprehensive structured intervention that can improve complex behavioral, psychological, and medical issues of patients (4). The European Society of Cardiology guidelines recommend aerobic exercise for CHF patients to reduce the risk of hospitalization (5).

Older patients have a higher need for exercise-based training because they have more difficulties in performing aerobic exercises than younger patients (6). Most studies have been carried out on Caucasian CHF patients. In China, heart failure is the leading cause of death in older patients (7) and Tai Chi (TC) (Tai Chi Chuan or Taijiquan) meditative programs are used for secondary prevention in heart failure patients (8). Functional electrical stimulation of lower limb muscles (FES) has improved Zung Self-Rating Depression scale scores (9), flow-mediated dilation (10), Beck Depression Inventory score (9), and 6-minute walking distance test results (11) in CHF patients. After doing a thorough literature search, one randomized trial with TC exercise (non-pharmacological intervention) on older CHF patients was found (12), and to our knowledge, only one study has evaluated the effect of FES on Chinese patients but not on older CHF patients (13). Therefore, the effect of physical activities on heart failure in older Chinese adults should be tested.

The objective of the study was to evaluate the effect of 12 weeks of FES of lower limb muscles and TC exercise in older Chinese adults with chronic systolic heart failure in terms of physical performance, quality of life, emotional stress, and cardiorespiratory functions.

## Material and Methods

### Ethics consideration and consent to participate

The study was registered in the Research Registry (UID No. 4474 dated January 1, 2015; http://www.researchregistry.com). The protocol (BLH/CMU/CL/15/14 dated December 26, 2014) was approved by the Beijing Luhe Hospital, Capital Medical University review board. An informed consent form was signed by all participating patients and/or their relatives (legally authorized persons) regarding the non-pharmacological interventions, pathology, and publications of the study in all formats (hard and/or electronic) irrespective of time and language. The study followed the law of China, Declaration of Helsinki (V 2008), and TREND (Improving the reporting quality of non-randomized evaluations of behavioral and public health interventions statement).

### Inclusion criteria

Patients aged 70 years and above who had right ventricular dysfunction, valvular disorders, combined systolic and diastolic heart failure, and had been previously hospitalized with exacerbation/decompensation of heart failure in the previous 3–6 months (prior to enrollment in the study) were included. Patients with stable CHF, i.e., only left ventricle dysfunction and with ejection fraction less than 50% (history of 2 years) were also included in the trial.

### Exclusion criteria

Patients with ejection fraction more than 50%, myocardial infarction within the preceding 3 months, major cardiac surgeries within the previous 6 months, cardiac arrest within the previous 6 months, who underwent cardiac resynchronization therapy within the previous 6 months, ventricular arrhythmias, peripartum cardiomyopathy, lower extremity amputation, and cognitive dysfunction were excluded from the study.

### Study design

A total of 1,084 patients were subjected to a non-randomized clinical trial. The decision of intervention(s) was taken by researchers according to the need and conditions of patients, e.g. patients who had an issue of muscle and/or joint pain were included for FES intervention, those who had no such issue were included for TCE intervention, those who did not want to take part in any exercise were included in the control group, and patients willing to adhere to both interventions were included in the FES & TCE group. Therefore, randomization was not possible.

### Sample size calculation

Weighted Mantel-Haenszel test was used to calculate the sample size. The propensity score analysis was defined as J strata and n denoted the total sample size and n_FES_ the sample size in the FES group. The data on each patient was comprised of the response variable (muscle and/or joint pain): x=1 (yes, FES group) and x=0 (no, TCE group) (14).

### Interventions

Patients who did not receive any kind of intervention were included in the control group (n=271). Patients who received 30 min a day (individually), 5-days/week FES for 12 weeks (9) were included in the FES group (n=271). Patients who received 1-h group classes of TC exercise twice weekly for 12 weeks (12) were included in the TCE group (n=271). Patients who received both interventions were included in the FES & TCE group (n=271). The same instructor was responsible for the TC exercises and the FES intervention.

### FES

Eight 60-mm adhesive electrodes were placed on the skin over the lower medial and upper lateral parts of the quadriceps muscle and over the lower and upper portions of the gastrocnemius muscles. A direct electrical current at 25 Hz was applied for 5 s. A 5-s interval was allowed between the two currents (9). RehaMove^®^ FES Cycling instrument (HASOMED GmbH, Germany) was used for all patients. One trained instructor (minimum of 3 years of experience) of the institute was the coordinator of the exercises.

### Tai Chi exercise

TC exercise included traditional warm-up exercise followed by weight shifting, arm swinging, gentle stretches (of legs, arms, spine, shoulders, and neck), visualization, and deep breathing. The exercise focuses on the relaxation of body and mind. Folding lounge chairs were provided for resting (Table 1) (15). One trained instructor (minimum of 3 years of experience) of the institute was the coordinator of the exercises. The exercise was performed early in the morning before breakfast.

**Table 1:** Tai chi exercise chart.

Exercises	Time of exercise
**Week 1**	
**Introduction**	
Tai chi philosophies	
Demonstration of form	
Expectations of participants	
Class format description	
**Warm-up exercises**	
Standing	
Drumming the body	6 min
Swinging to connect the lungs and kidneys	3 min
Washing the body with ‘*qi*'	3 min
Breathing and standing meditation	3 min
Sitting	
Shoulder/neck stretching	6 min
Leg/arm stretching	3 min
Breathing and sitting meditation	6 min
	30 min total
**Weeks 2–5**	
**Repetition of warm-up exercises**	
**Tai chi movements**	
Raising the power	10 min
Withdraw and push	5/side
**Weeks 6–9**	
**Repetition of warm-up exercises and Tai chi movements**	
Brush knee twist step	5/side
Grasp sparrows tail	5/side
**Weeks 10–12**	
**Repetition of all exercises of 9 weeks**	
Wave hands like clouds	10 min

These exercises were performed in the early morning before breakfast.

### Primary outcome measures

#### Kansas City Cardiomyopathy Questionnaire

This 23-item questionnaire evaluates the physical and social quality of life of patients. The score ranges from 0 to 100, with 0–25 indicating severe symptoms and complete disability, 26–50 indicating moderate symptoms, 51–75 indicating fair symptoms, and 76–100 indicating no symptom and no disability (16).

#### Zung Self-Rating Depression Scale

This scale was used to screen emotional stress. It is a 20-item questionnaire regarding the psychological and somatic symptoms of patients. Each question has five grades. The total score is graded as 0–25: no depression, 26–50: fair depression, 51–75: moderate depression, and 76–100: severe depression (17).

#### Beck Depression Inventory

This scale has six subscales and a grading system for 10 symptoms. The total score is 60. Higher scores indicate more depression (18).

#### Endothelium-dependent brachial artery flow-mediated dilatation

The right brachial artery 2.1 cm above the elbow was selected for measurement of flow-mediated dilatation evaluation. Patients were instructed to lie down for 15 min before the scan. An electrocardiogram was taken before the scan. The arterial diameter was measured by color Doppler (GE Healthcare, USA) at the peak of the R wave in the electrocardiogram. The mean of five observations was considered for analysis. A scan after deflation of the cuff was performed after a 100-s interval. There was a 2-min interval between observations. Flow-mediated dilatation was measured according to the following equation (19,20): [Flow-mediated dilatation = branchial arterial diameter – branchial arterial diameter after deflation of cuff / branchial arterial diameter]. One radiologist (minimum of 3 years of experience) of the Institute performed and evaluated the scans.

### Secondary outcome measures

#### Timed “Up & Go*”* measurement

The time required for the patient to stand up from a folding lounge chair, walk 3 m, turn, walk back, and sit down again in the chair was recorded (15).

#### Peak oxygen consumption measurement

A bicycle ramp protocol was used to evaluate peak oxygen consumption. The highest oxygen consumption achieved during the last 30 s of peak exercise was considered to be peak oxygen consumption.

All patients were evaluated for outcome measures at baseline and after 12-weeks of interventions. The evaluators of the institute involved in the study were blinded regarding the interventions. Any worst outcome was considered an adverse effect as per institutional guidelines of a non-pharmacological study.

### Statistical analysis

Continuous variables are reported as means±SD and discrete characteristics are reported as a number (percentage). SPSS version 25 (IBM Corporation, USA) was used for statistical analysis. Repeated measures of multivariate analysis of variance (RM-MANOVA) followed by Tukey’s *post hoc* test (considering critical value [*q*]>3.633 as significant) was used to compare continuous data between groups. The chi-squared Independence test was used for discrete data (19). RM-MANOVA test (15) followed by Tukey’s *post hoc* test (considering critical value [*q*]>3.633 as significant) was used for statistical comparison between baseline and after 12 weeks of interventions within a group. All data were considered significant at P<0.05.

## Results

### Enrollment

**Figure 1.**
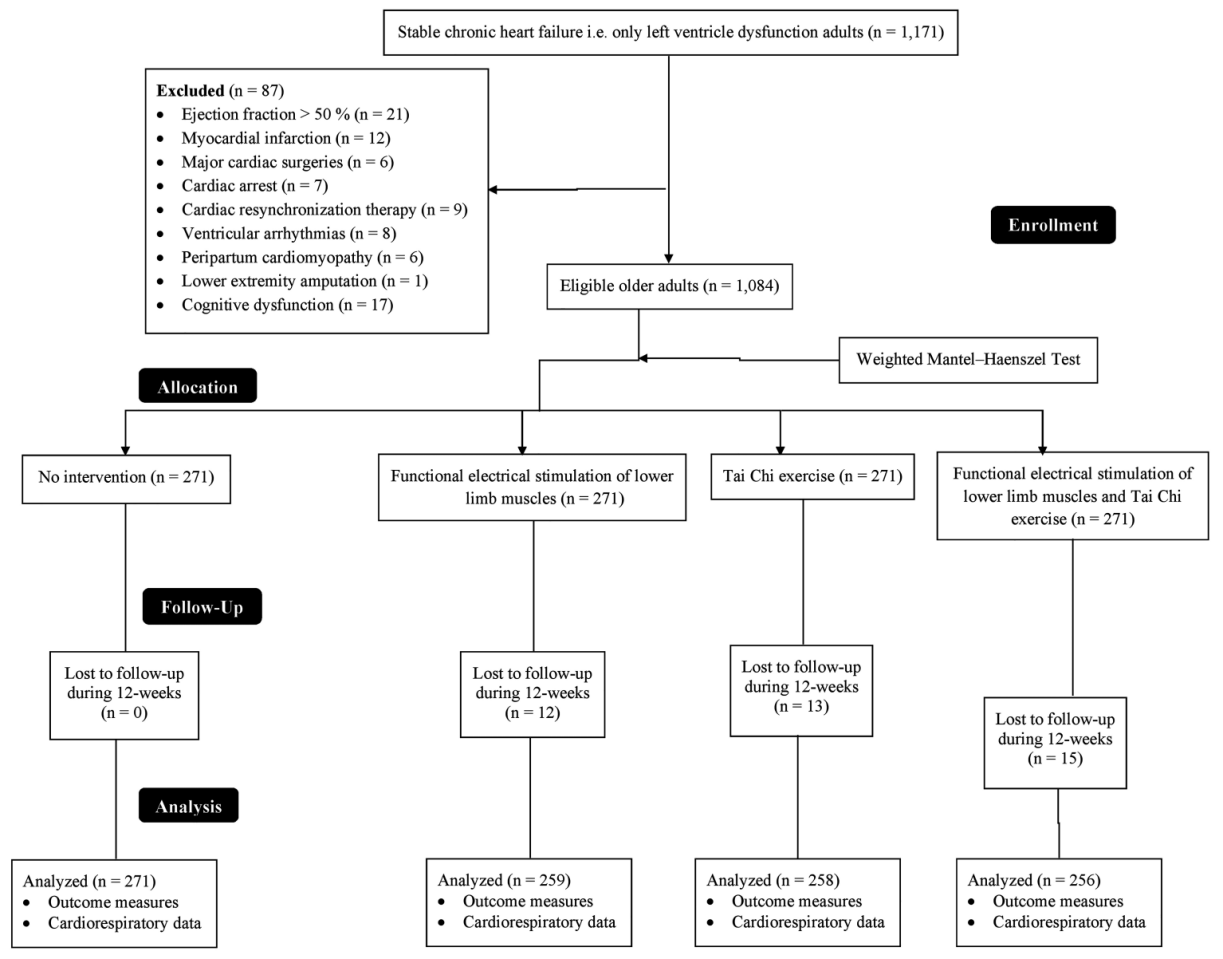
Flow diagram of the study.

A total of 1,171 patients with stable CHF and age ≥70 years were available at the Beijing Luhe Hospital, Capital Medical University, China and the referring hospitals from January 2, 2015 to July 1, 2017. Among them, 21 patients had ejection fraction greater than 50%, 12 patients had myocardial infarction, six patients had undergone major cardiac surgeries, seven patients had cardiac arrest problems, nine patients were planned for cardiac resynchronization therapy, eight patients had ventricular arrhythmias, six patients had peripartum cardiomyopathy, one patient had amputation of a lower extremity, and 17 patients had cognitive dysfunction. Therefore, they were not included. A total of 1,084 patients were enrolled in the study (Figure 1).

### Demographic characteristics and clinical parameters

All the enrolled patients had moderate symptoms of depression at the time of the enrollment; 56% were male and 44% female. The mean blood pressure was above normal for systolic heart failure patients (136±8 mmHg); diastolic blood pressure also was elevated (88–90 mmHg), above the goal of blood pressure of 120–130/80 mmHg. Heart rate was an average of 74–84 beats per minute. The other demographic and clinical features of the enrolled patients are presented in Supplementary Table S1. There was no significant difference among the groups regarding demographic characteristics and clinical parameters at the time of enrollment (P≥0.05 for all). Twelve participants from the FES group, 13 from the TCE group, and 15 from the FES & TCE group were lost during the follow-up of 12-weeks. The data of the remaining participants were used in the statistical analysis.

Compared to the control group, FES, TC exercise, and FES & TCE exercise were not effective to improve Zung Self-Rating Depression scale (P=0.79), Beck Depression Inventory score (P=0.114), and the Timed “Up & Go” time (Supplementary Table S2). Compared to the control group, FES & TCE had decreased systolic blood pressure (P<0.0001, q=4.57) and diastolic blood pressure (P=0.006, q=3.88). Also, FES, TCE, and FES & TCE had improvement of outcome measures and cardiorespiratory data at the end of 12 weeks of intervention compared to baseline (Supplementary Table S3). No adverse effect was reported during the 12 weeks of the study and during the follow-up period in all groups.

**Figure 2.**
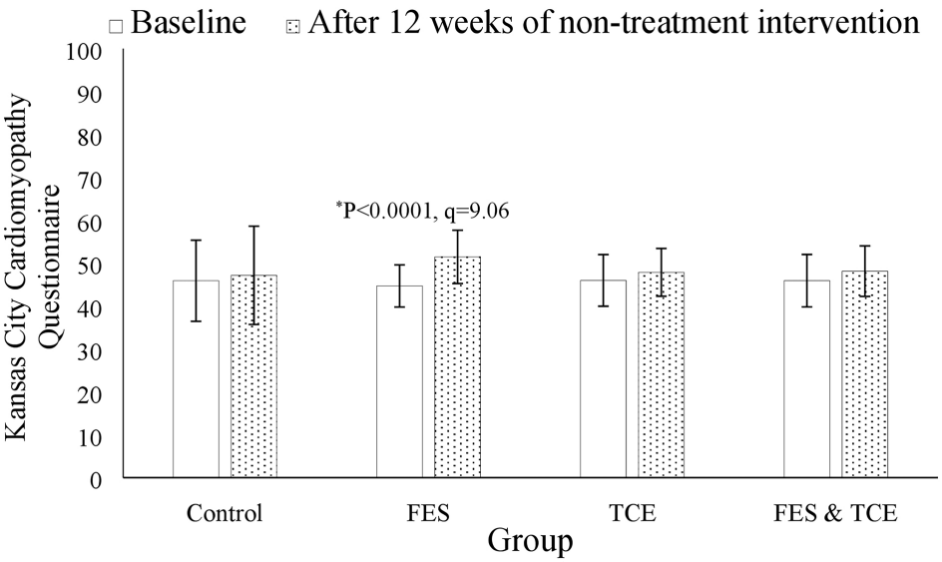
Effects of interventions on the Kansas City Cardiomyopathy Questionnaire scores. RM-MANOVA followed by Tukey’s *post hoc* test was used for statistical analysis. Data are reported as means±SD at baseline and after 12 weeks of non-pharmacological interventions. *P<0.05 compared to the control group. FES: functional electrical stimulation of lower limb muscles; TCE: Tai Chi exercise.

**Figure 3.**
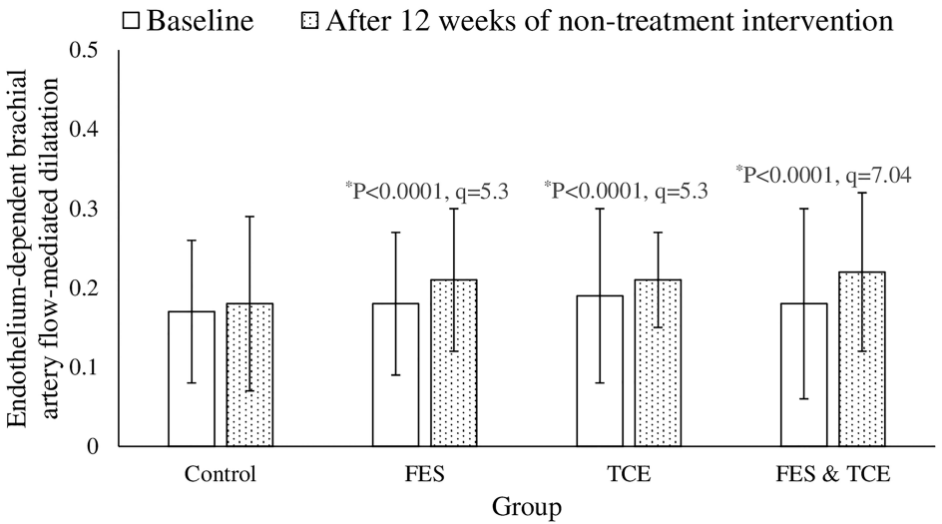
Effects of non-pharmacological interventions on endothelium-dependent brachial artery flow-mediated dilatation. RM-MANOVA followed by Tukey’s *post hoc* test was used for statistical analysis. Data are reported as means±SD at baseline and after 12 weeks of intervention. *P<0.05 compared to the control group. FES: functional electrical stimulation of lower limb muscles; TCE: Tai Chi exercise.

**Figure 4.**
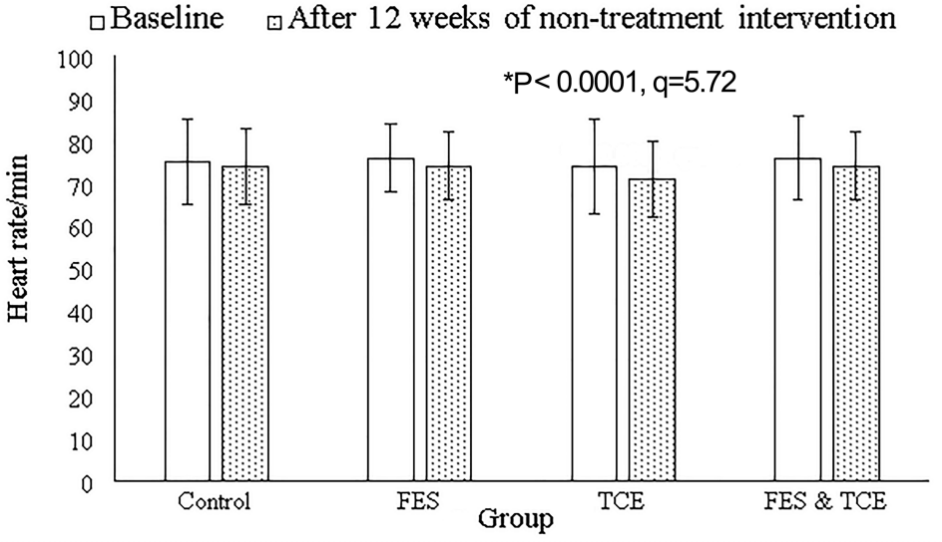
Effects of non-pharmacological interventions on heart rate. RM-MANOVA followed by Tukey’s *post hoc* test was used for statistical analysis. Data are reported as means±SD at baseline and after 12 weeks of intervention. The evaluators of the institute were blinded regarding the interventions. *P<0.05 compared to the control group. FES: functional electrical stimulation of lower limb muscles; TCE: Tai Chi exercise.

**Figure 5.**
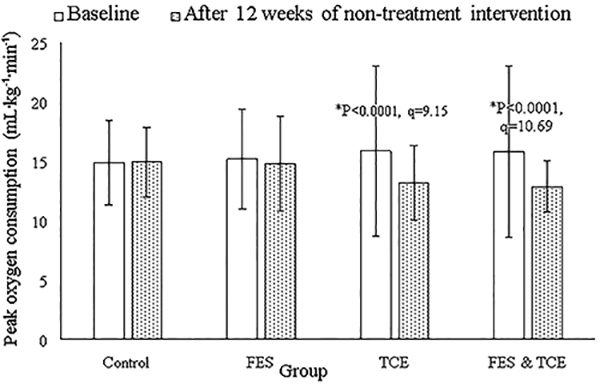
Effects of non-pharmacological interventions on peak oxygen consumption. RM-MANOVA followed by Tukey’s *post hoc* test was used for statistical analysis. Data are reported as means±SD at baseline and after 12 weeks of intervention. *P<0.05 compared to the control group. FES: functional electrical stimulation of lower limb muscles; TCE: Tai Chi exercise.

Compared to the control group, only FES had an increased KCCQ score (P<0.0001, q=9.06, Figure 2). At the end of the non-pharmacological interventions, FES (P=0.0001, q=5.02) and FES & TCE groups (P<0.0001, q=5.87) had improved endothelium-dependent brachial artery flow-mediated dilatation (Figure 3). Compared to the control group, heart rate was decreased in the TCE group (P<0.0001, q=5.72, Figure 4) and peak oxygen consumption was decreased in the TCE (P<0.0001, q=9.15) and FES & TCE groups (P<0.0001, q=10.69, Figure 5).

### Cost

The cost for TCE was 6,010±55 ¥/patient, for FES, 5,500±75 ¥/patient, and for FES & TCE, 11,000±100 ¥/patient.

## Discussion

### Physical and social quality of life

FES, TCE, and FES & TCE were found to be safe, effective, and affordable for older CHF Chinese patients to improve their physical and social quality of life and cardiorespiratory functions compared to the control group. Heart failure in older adults has high morbidity and mortality and the use of the first clinical intervention for management is unclear (15). Pharmacological therapies for depression may improve the quality of life of older CHF adults but do not guarantee better results (21). Exercise is beneficial in older CHF adults (3,15,22). KCCQ is an important and valid tool to evaluate long-term event-free survival in these patients (23).

### Endothelium-dependent brachial artery flow-mediated dilatation

Compared to the control group, endothelium-dependent brachial artery flow-mediated dilatation was improved by FES (P<0.0001, q=5.3), TCE (P<0.0001, q=5.3), and FES & TCE (P<0.0001, q=7.04). Endothelium vasoconstriction increases mortality risk in CHF older adults (24). It is obvious that TCE improved endothelium vasodilatation. However, FES promotes nitric oxide degradation, and the free available oxygen in blood (10) results in vasodilatation. The results of the study were consistent with previous studies (9–11). FES is important for CHF patients who cannot adhere to TCE.

### Cardiorespiratory data

Eighty-three participants in the TCE group, 71 in the FES group, and 78 in the control group used beta-blockers. However, TCE reduced heart rate and peak oxygen consumption of patients. Reduction in peak oxygen consumption is due to a reduction in heart rate (15). Depression severity, autonomic nervous dysfunction, and low quality of life in CHF patients are correlated with increased heart rate (25). TCE harmonizes body and mind (26). The results of the study were in line with previous studies (12,15,27). TCE may provide benefits as a mood stabilizer and regulation of the autonomic nervous system in older CHF adults.

### Failure outcome measures

FES, TCE, and FES & TCE were not effective to improve Zung Self-Rating Depression Scale score, Beck Depression Inventory score, and the Timed “Up & Go”. The results were not consistent with previous studies (9,11) but were consistent with a randomized clinical trial (12). The possible justification for such results is that our study enrolled higher numbers of patients with New York Heart Association Functional Classification (NYHA) I and II. While FES exerts a greater beneficial effect on the clinical and neurohormonal status of NYHA III and IV patients (11), the benefits may be smaller for neuropsychiatric parameters.

### Cost

FES and TCE were performed at affordable rates. Heart failure imposes a considerable financial burden on patients (28) for treatment, pathology, and radiology (29).

### Limitations

Although there are no studies that compared TCE and FES in older CHF Chinese adults, the current study has several limitations, for example, the B-type natriuretic peptide data was not available for the study population. Although aerobic exercise is the ‘gold standard' for cardiac rehabilitation, the study did not include an aerobic exercise group (positive control group) because the participants were unable to perform aerobic exercises. The study lacked randomization, thus biased results may have occurred, which is a big threat to the validity of the study. Randomization was not possible in this study because the non-pharmacological intervention was selected by the clinician according to need and conditions of the patients. Analyses of gender, age, duration of heart failure, and body composition were not performed.

In conclusion, FES of lower limb muscles and TCE can recover the physical and social quality of life and cardiorespiratory functions of older Chinese CHF adults. FES is also a good option for older adults who are unable to perform TCE.

## Supplementary Material

Click here to view [pdf].

## References

[1] Zhang Y, Zhang J, Butler J, Yang X, Xie P, Guo D (2017). Contemporary epidemiology, management, and outcomes of patients hospitalized for heart failure in China: results from the China heart failure (China-HF) registry. J Card Fail.

[2] Okita K, Kinugawa S, Tsutsui H (2013). Exercise intolerance in chronic heart failure--skeletal muscle dysfunction and potential therapies. Circ J.

[3] O'Connor CM, Whellan DJ, Lee KL, Keteyian SJ, Cooper LS, Ellis SJ (2009). Efficacy and safety of exercise training in patients with chronic heart failure: HF-ACTION randomized controlled trial. JAMA.

[4] Smart NA, Dieberg G, Giallauria F (2013). Functional electrical stimulation for chronic heart failure: a meta-analysis. Int J Cardiol.

[5] Ponikowski P, Voors AA, Anker SD, Bueno H, Cleland JG, Coats AJ (2016). 2016 ESC Guidelines for the diagnosis and treatment of acute and chronic heart failure: the Task Force for the diagnosis and treatment of acute and chronic heart failure of the European Society of Cardiology (ESC). Developed with the special contribution of the Heart Failure Association (HFA) of the ESC. Eur J Heart Fail.

[6] Yamamoto S, Matsunaga A, Ishida T, Misawa K, Yamazaki S, Higuchi S (2017). Exercise-based cardiac rehabilitation for elderly patients with coronary artery disease. JSM Physical Med Rehabil.

[7] Guo Y, Lip GY, Banerjee A (2013). Heart failure in East Asia. Curr Cardiol Rev.

[8] Sun J, Buys N, Jayasinghe R (2014). Effects of community-based meditative Tai Chi programme on improving quality of life, physical and mental health in chronic heart-failure participants. Aging Ment Health.

[9] Parissis J, Karavidas A, Farmakis D, Papoutsidakis N, Matzaraki V, Arapi S (2015). Efficacy and safety of functional electrical stimulation of lower limb muscles in elderly patients with chronic heart failure: a pilot study. Eur J Prev Cardiol.

[10] Deftereos S, Giannopoulos G, Raisakis K, Kossyvakis C, Kaoukis A, Driva M (2010). Comparison of muscle functional electrical stimulation to conventional bicycle exercise on endothelium and functional status indices in patients with heart failure. Am J Cardiol.

[11] Karavidas A, Parissis JT, Matzaraki V, Arapi S, Varounis C, Ikonomidis I (2010). Functional electrical stimulation is more effective in severe symptomatic heart failure patients and improves their adherence to rehabilitation programs. J Card Fail.

[12] Yeh GY, McCarthy EP, Wayne PM, Stevenson LW, Wood MJ, Forman D (2011). Tai chi exercise in patients with chronic heart failure: a randomized clinical trial. Arch Intern Med.

[13] Chen D, Yan T, Li G, Li F, Liang Q (2014). Effect of functional electrical stimulation on lower limb motor function and magnetic resonance diffusion tensor imaging in early stroke patients. [ in Chinese]. Chinese Med J.

[14] Jung SH, Chow SC, Chi EM (2007). A note on sample size calculation based on propensity analysis in nonrandomized trials. J Biopharm Stat.

[15] Yeh GY, Wood MJ, Wayne PM, Quilty MT, Stevenson LW, Davis RB (2013). Tai chi in patients with heart failure with preserved ejection fraction. Congest Heart Fail.

[16] Joseph SM, Novak E, Arnold SV, Jones PG, Khattak H, Platts AE (2013). Comparable performance of the Kansas City Cardiomyopathy Questionnaire in patients with heart failure with preserved and reduced ejection fraction. Circ Heart Fail.

[17] Suzuki T, Shiga T, Kuwahara K, Kobayashi S, Suzuki S, Nishimura K (2014). Impact of clustered depression and anxiety on mortality and rehospitalization in patients with heart failure. J Cardiol.

[18] Lahlou-Laforet K, Ledru F, Niarra R, Consoli SM, PANIC Investigators (2015). Validity of Beck Depression Inventory for the assessment of depressive mood in chronic heart failure patients. J Affect Disord.

[19] Naidu OA, Rajasekhar D, Latheef SA (2011). Assessment of endothelial function by brachial artery flow-mediated dilatation in microvascular disease. Cardiovasc Ultrasound.

[20] Atkinson G, Batterham AM (2013). The percentage flow-mediated dilation index: A large-sample investigation of its appropriateness, potential for bias and causal nexus in vascular medicine. Vasc Med.

[21] Liguori I, Russo G, Curcio F, Sasso G, Della-Morte D, Gargiulo G (2018). Depression and chronic heart failure in the elderly: an intriguing relationship. J Geriatr Cardiol.

[22] Piepoli MF, Conraads V, Corra U, Dickstein K, Francis DP, Jaarsma T (2011). Exercise training in heart failure: from theory to practice. A consensus document of the Heart Failure Association and the European Association for Cardiovascular Prevention and Rehabilitation. Eur J Heart Fail.

[23] Parissis JT, Nikolaou M, Farmakis D, Paraskevaidis IA, Bistola V, Venetsanou K (2009). Self-assessment of health status is associated with inflammatory activation and predicts long-term outcomes in chronic heart failure. Eur J Heart Fail.

[24] Kishimoto S, Kajikawa M, Maruhashi T, Iwamoto Y, Matsumoto T, Iwamoto A (2016). Endothelial dysfunction and abnormal vascular structure are simultaneously present in patients with heart failure with preserved ejection fraction. Int J Cardiol.

[25] von Kanel R, Saner H, Kohls S, Barth J, Znoj H, Saner G (2009). Relation of heart rate recovery to psychological distress and quality of life in patients with chronic heart failure. Eur J Cardiovasc Prev Rehabil.

[26] Lin GM, Tzeng BH (2011). Yin and yang of tai chi exercise. Arch Intern Med.

[27] Gu Q, Wu SJ, Zheng Y, Zhang Y, Liu C, Hou JC (2017). Tai Chi exercise for patients with chronic heart failure: a meta-analysis of randomized controlled trials. Am J Phys Med Rehabil.

[28] Callender T, Woodward M, Roth G, Farzadfar F, Lemarie JC, Gicquel S (2014). Heart failure care in low- and middle-income countries: A systematic review and meta-analysis. PLoS Med.

[29] Burch D (2014). Heart failure: gaps in knowledge and failures in treatment. PLoS Med.

